# Linking prokaryotic community composition to carbon biogeochemical cycling across a tropical peat dome in Sarawak, Malaysia

**DOI:** 10.1038/s41598-021-81865-6

**Published:** 2021-03-19

**Authors:** Simon Peter Dom, Makoto Ikenaga, Sharon Yu Ling Lau, Son Radu, Frazer Midot, Mui Lan Yap, Mei-Yee Chin, Mei Lieng Lo, Mui Sie Jee, Nagamitsu Maie, Lulie Melling

**Affiliations:** 1 Sarawak Tropical Peat Research Institute, Lot 6035, Kuching-Samarahan Expressway, 94300 Kota Samarahan, Sarawak Malaysia; 2 Department of Food Science, Faculty of Food Science and Technology, Universiti Putra Malaysia, 43400 Serdang, Selangor Malaysia; 3 Research Field in Agriculture, Agriculture Fisheries and Veterinary Medicine Area, Kagoshima University, 1-21-24, Korimoto, Kagoshima, 890-0065 Japan; 4 School of Veterinary Medicine, Kitasato University, Towada, Aomori 034-8628 Japan

**Keywords:** Metagenomics, Microbial ecology

## Abstract

Tropical peat swamp forest is a global store of carbon in a water-saturated, anoxic and acidic environment. This ecosystem holds diverse prokaryotic communities that play a major role in nutrient cycling. A study was conducted in which a total of 24 peat soil samples were collected in three forest types in a tropical peat dome in Sarawak, Malaysia namely, Mixed Peat Swamp (MPS), Alan Batu (ABt), and Alan Bunga (ABg) forests to profile the soil prokaryotic communities through meta 16S amplicon analysis using Illumina Miseq. Results showed these ecosystems were dominated by anaerobes and fermenters such as Acidobacteria, Proteobacteria, Actinobacteria and Firmicutes that cover 80–90% of the total prokaryotic abundance. Overall, the microbial community composition was different amongst forest types and depths. Additionally, this study highlighted the prokaryotic communities’ composition in MPS was driven by higher humification level and lower pH whereas in ABt and ABg, the less acidic condition and higher organic matter content were the main factors. It was also observed that prokaryotic diversity and abundance were higher in the more oligotrophic ABt and ABg forest despite the constantly waterlogged condition. In MPS, the methanotroph *Methylovirgula ligni* was found to be the major species in this forest type that utilize methane (CH_4_), which could potentially be the contributing factor to the low CH_4_ gas emissions. *Aquitalea magnusonii* and *Paraburkholderia oxyphila*, which can degrade aromatic compounds, were the major species in ABt and ABg forests respectively. This information can be advantageous for future study in understanding the underlying mechanisms of environmental-driven alterations in soil microbial communities and its potential implications on biogeochemical processes in relation to peatland management.

## Introduction

Soil microorganisms play central roles in nutrient transformations in mediating soil organic matter decomposition and nutrient cycling in the forest ecosystems^1^. These microorganisms have dual roles in the soil, as an agent for the decomposition of plant residues and are a labile pool of nutrients^2^. The primary drivers in determining the microbial community composition in soil are pH, temperature, soil moisture, organic matter content, nutrient availability and biotic interactions, with increased microbial activity and abundance in hotspots such as litter, deadwood and rhizospheres^3^. These plant biomasses provide the most important C sources for forest soil microbes. Although fungi are widely-studied and are considered as the main decomposers in forest soils of many forest types due to their ability to produce a wide-range of extracellular enzymes that enables them to effectively degrade recalcitrant component of plant biomass^4^, recent findings have indicated that bacteria also commonly possess genes encoding plant cell wall-degrading enzymes^5^. In fact, bacteria incorporated relatively more cellulose-derived C than that incorporated by fungi^6^. Therefore, bacterial involvement in decomposition appears to be a relatively common trait.

Tropical peatland is a globally significant carbon reservoir, where peat is accumulated from organic matter mostly of plant materials due to a higher rate of deposition and a lower rate of decomposition because of waterlogged anaerobic condition^7–9^. Aside from thick leaf litters, peat in the tropics are largely composed of coarse woody material from fallen trees, branches and thick dead root mats^10^. The decomposition rate of coarse woody materials is slower due to high lignin content and low surface-area-to-volume ratio that limits even anaerobic decomposition^11^. Lignin and other unsaturated acid-insoluble material such as tannins and humic substances are known as aromatics that are more recalcitrant, whereas acid-hydrolysable polysaccharides are carbohydrates that are labile^12^. In addition, aromatics are known to inhibit anaerobic decomposition^13^.

Malaysia’s tropical peatlands cover about 2.7 × 10^4^ km^2^ of the total land area, in which most of these areas are located in Sarawak and Sabah with an area of approximately 1.7 × 10^4^ km^2^ ^14^. In Sarawak, a distinct transition in the structure of the forest vegetation can be observed from the edge to the center of a peat dome. It is characterized by an uneven-canopied high forest from the edge of the swamp to zones of lower tree height, decreased tree girth, thicker leaves and lower tree species richness towards the centre of the swamp. The characterization based on this phasic community zonation has classified six forest types namely Mixed Peat Swamp, Alan Batu, Alan Bunga, Padang Alan, Padang Selunsor and Padang Keruntum^15–17^. However, the three main forest types are Mixed Peat Swamp (MPS), Alan Batu (ABt) and Alan Bunga (ABg) forests^18^. On a tropical peat dome, MPS forests are predominantly located at the edge of the dome and adjacent to the main river. Due to the fact that it lies at a lower elevation, it receives nutrients carried by the water flowing downslope. Consequently, MPS is of higher soil fertility and humification level, which is indicated by richer tree species composition. The structure and physiognomy of MPS are highly similar to a mineral soil lowland dipterocarp rain forest with abundance of epiphytes and climbers. ABt forests are generally located at the slope of a tropical peat dome with the highest water flow through the area. The hydrological movement creates an austere environment and makes it unique as trees experience physiological adaptation by having bigger buttresses and extensive root system. ABg forests are located towards the tip of the peat dome and trees are also found to have extensive roots^18,19^. All these environmental conditions along with other factors such as aboveground vegetation^20^, the quality and quantity of the litter^21^ and soil depth^22,23^ play important role in shaping soil microbial communities. In a previous study on these three peat forest types, the highest decomposability rate based on soil C flux, is in the order ABg > ABt > MPS^19^. Soil C flux is a method commonly used to measure decomposition of peat^19^. However, fertility of peat soil in terms of humification level and availability of nutrients to plants is in the reversed order MPS > ABt > ABg. A study conducted by Millard and Singh^24^, suggested that the quality and composition of organic matter play a huge impact on the microbial diversity especially bacteria. Therefore, it is important to understand the effects of forest types and soil depth on soil microbial community composition as it could provide insight regarding the community structure and function relationship and relative contributions of various microbial groups to SOM transformation for better peatland management.

Advancement in molecular methods has allowed higher resolution assessment of microbial compositions in complex tropical peatland ecosystem. There are only a few studies conducted using next-generation sequencing (NGS) technology to look into bacterial community composition in tropical peatlands. Such studies have been conducted in tropical peat swamp forest in Thailand^25^, Indonesia^26^, Brunei^27^, and Peninsular Malaysia^28^. Their results demonstrated that Acidobacteria and Proteobacteria are the two most dominant phyla in the tropical peat swamp forests. Interestingly, Tripathi et al.^27^, confirmed that geologically distinct environments such as the tropical peat swamp forests and heath forest are about as microbiologically diverse or more than tropical mixed dipterocarp forests, whilst Too et al.^28^ reported that depth but not tree species influences microbial community structure in a tropical peat swamp forest in Peninsular Malaysia.

The study by Sangok et al.^19,29^ has characterized SOM decomposability and the accumulation rate of tropical peat soils from the different forest types in Maludam National Park, Sarawak. It is hypothesized that the microbial community composition is reflected by the peat soil decomposition level of each forest type. Thus, the objective of this study is to profile the prokaryotic community variation in the three common forest types across a peat dome using the high-throughput sequencing to relate to the carbon biogeochemical cycling in a tropical peat dome.

## Materials and methods

### Study site and soil sampling

The Maludam National Park (1°31′ N, 111°08′ E) located at Betong Division, Sarawak is the largest single patch of peat swamp forest in Sarawak, covering an area of 430 km^2^ and was gazetted in May 2000^18^. Prior to the designation as the Maludam National Park, the forest has undergone selective logging, where trees with DBH of more than 45 cm have been harvested. The selective logging activity was only stopped in the 1990s^30^. The area has mean annual precipitation of 3182 mm and mean annual temperature of 27.2 °C during 2017–2018. Generally, a distinct shift in the structure of forest vegetation can be observed from the edge to the center of the peat dome. Across a peat dome, the peat swamp forest can be characterized into six phasic community types and we studied the three most common forest types: phasic community 1 (Mixed Peat Swamp forest) is a *Gonystylus-Dactylocladus-Neoscortechinia* association, phasic community 2 (Alan Batu forest) is a *Shorea albida-Gonystylus-Stenonurus* association and phasic community 3 (Alan Bunga forest) is a *S. albida* consociation^15^.

**Figure 1:**
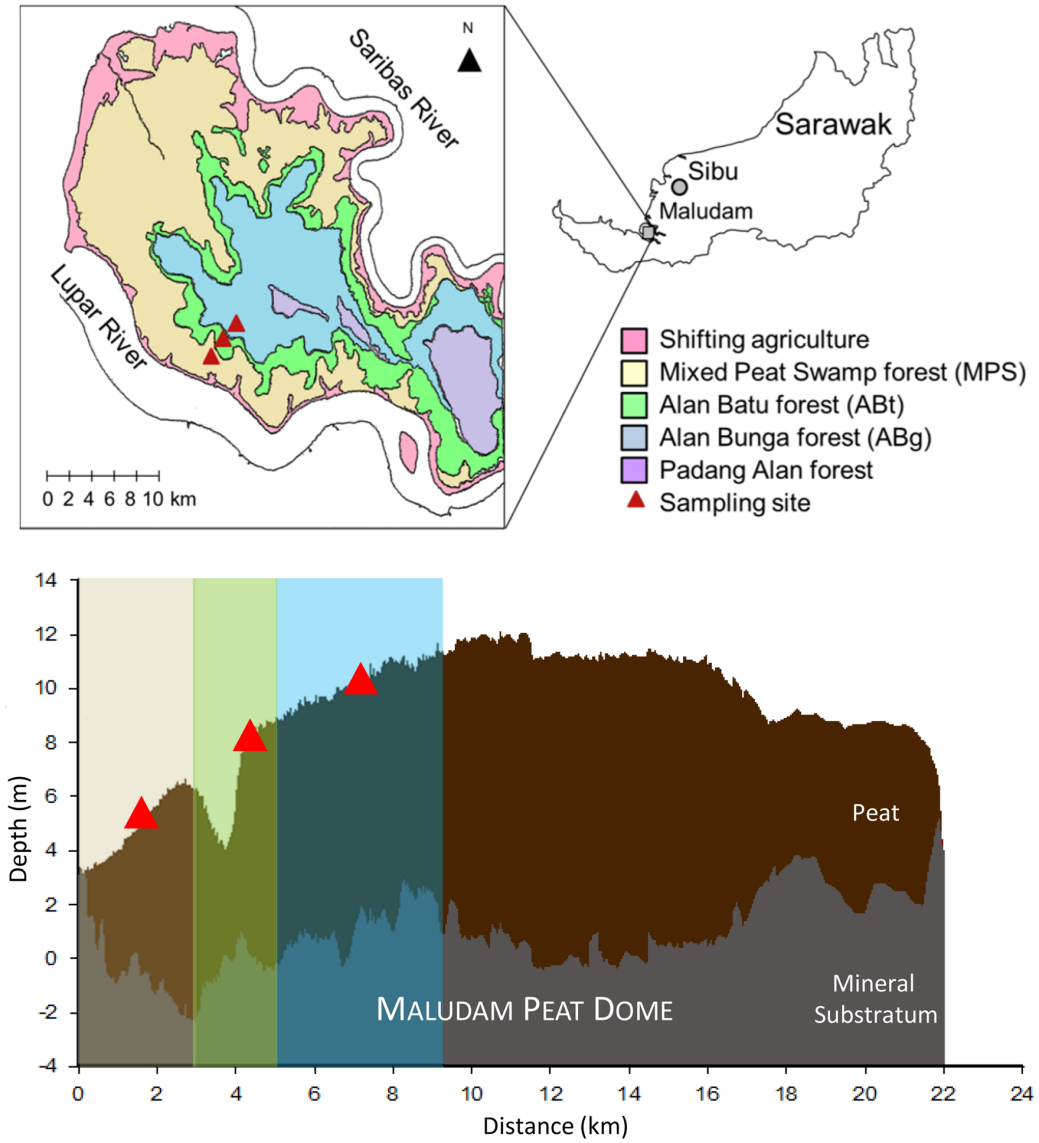
Map of Maludam National Park showing the location of study sites across the Maludam peat dome in Betong, Sarawak, Malaysia. MPS: site located at the edge of the dome, adjacent to the main river and at a lower elevation; ABt: site located at the slope of a peat dome with high water flow through the area; ABg; site located towards the tip of a peat dome. Adaptation of maps and diagram from Melling^18^ and Sangok et al.^19^.

Field sampling was carried out in April, October, December 2017 and February 2018. Peat soil samples were collected using a peat auger (Eijkelkamp, The Netherlands) from three forest types namely Mixed Peat Swamp forest (MPS), Alan Batu forest (ABt), and Alan Bunga forest (ABg) (Fig. 1). Samples were taken at two depths; 0–20 cm (T) and 30–50 cm (B) from the soil column. Mid-section (20–30 cm) of the soil was excluded from the analysis to clearly distinguish between the top and bottom layers. Composite soil samples (2.0 kg) were collected randomly at each forest type. Soil samples were placed in polyethylene zipper bags and the air inside the bags were eliminated as much as possible. For transportation to the laboratory, soil samples were stored in cooler box (with ice packs) and kept away from direct sunlight. A total of 24 peat soil samples were collected from the three forest types. Soil samples were stored at − 20 °C and 4 °C prior to DNA extraction and soil chemical analyses, respectively.

### Water table measurement

Water table (WT) measurement was taken by measuring the water level in the perforated PVC pipes, which were installed previously at each forest type. Water table is calculated as WT (cm) = H1 – H2, where H1 is the height from the top of the pipe until the water surface inside the pipe and H2 is the height from the top of the pipe until ground surface outside the pipe. The measurements were taken from three different sides of the pipe circumference to obtain an average reading at each sampling site.

### Soil chemical analyses

Soil pH was determined by weighing 5.0 g of fresh peat soil sample into a 50 ml Falcon tube which was then added with 12.5 ml of ultrapure water (UPW) based on the 2:5 (w/v) soil to water ratio. The solution was placed on an orbital shaker SSL1 (Stuart, UK) at 300 rpm for 1 h and left overnight to allow the suspension to settle down and formed an aqueous layer. The pH reading was taken using a pH meter Metrohm 827 (Metrohm, Switzerland). Loss on ignition (LOI) was determined by weighing approximately 2.0 g of the peat soil sample and analyzed using thermo gravimetric analyzer TGA 701 (Leco, USA). LOI analysis is used to determine the organic matter content in soil by measuring weight loss after burning^31^. Total nitrogen (N) and total carbon (C) was determined by using a TruMac CN analyzer (Leco, USA) and from those values, the C/N ratio was calculated. The C/N ratio is a method to determine the state of organic matter decomposition^32^ as there is a correlation between C/N ratios and humification degree. As for the pyrophosphate solubility index (PSI), 0.5 g of peat soil samples were added with 50 ml of 0.025 M of sodium pyrophosphate (NA_4_P_2_O_7_.10H_2_O) and mixed-well using a shaker for 18 h before filtering the mixture using Whatman Filter Paper No. 5 (Whatman, UK). The filtered solution was then diluted with distilled water and measured using an ultraviolet visible spectrometer UV/VIS Lambda 25 (Perkin Elmer, USA). This method is a colorimetric estimation of the degree of humification of organic soil by measuring the color intensity of an extract obtained by treating the peat sample with sodium pyrophosphate solution which is able to extract the dark colored substance quantitatively^33^.

### Total DNA extraction and 16S rRNA gene amplification

Total DNA was extracted from approximately 2.0 g of fresh peat soil sample using the protocols in Lau^34^. The quality of DNA was examined on 1% agarose gel and the concentration was measured with NanoPhotometer P-360 (IMPLEN, Germany). The DNA samples concentration was ensured to be more than 10 ng/µl (Supplementary Table S1). A PCR amplification was carried out using universal primers 341F-GC (5′-CCTACGGGAGGCAGCAG-3′) and 907R (5′-CCGTCAATTCMTTTGAGTTT-3′)^35^, targeting the V4 hypervariable region in the bacterial 16S rRNA gene. Amplification was done in 30 µl reaction volumes containing 2.25 U of Amplitaq Gold DNA polymerase (Applied Biosystems, USA), 0.2 mM dNTPs mixture (Vivantis Technologies, Malaysia), 2.0 mM MgCl_2_ (Promega, USA), 3 µl of 10X AmpliTaq buffer (Applied Biosystems, USA), 0.5 mM of each primer and 1.15–6.39 ng of soil DNA. The PCR procedure was conducted using a MasterCycler Nexus G2 (Eppendorf, Germany) under the following protocols; 94 °C for 3 min (initial denaturation), 25 cycles of 1 min at 94 °C (denaturation), 1 min at 54 °C (annealing of primers) and 2 min at 72 °C (extension), and at 72 °C for 10 min (final extension). PCR products were run on the 2.0% (w/v) gel and viewed using the Molecular Imager Gel Doc XR system (BioRad, USA).

### Illumina sequencing and sequence processing

Samples were purified using a gel extraction kit NucleoSpin Gel and PCR Clean-up (Macherey–Nagel, Germany) according to the manufacturer instruction prior to sequencing. All 24 purified samples were sent to Seibutsu Giken (Japan) for sequencing. Initially, a library was prepared from the purified samples using a 2-step tailed PCR method and the concentration of the prepared library was measured using Synergy H1 and QuantiFluor dsDNA System while its quality was confirmed using Fragment Analyzer and ds DNA 915 Reagent Kit (Advanced Analytical Technologies, Inc.). After that, the sequencing analysis was performed using Illumina Miseq under the condition of 2 × 300 bp. Using Fastq_barcode_splitter of Fastx toolkit, only sequences that matched exactly with the primer were extracted and sequences with low quality score (< 20) were removed leaving a final base pair length of 150 or less. Pre-processed sequences were then analyzed in Quantitative Insights into Microbiology Ecology (QIIME)^36^ and clustered into Operational Taxonomic Units (OTUs) using Greengenes database at 97% similarity. The alpha diversity indices such as Shannon-index, Chao1, Observed species, and Phylogenetic Diversity (PD) whole tree were also determined using QIIME script. Observed species, Shannon-index and Chao1 were generated based on OTU counts, while PD whole tree quantified the branch diversity of the phylogenetic tree^37–39^. The Shannon-index is an estimator of species richness and species evenness with more weight on species richness^40,41^ whereas, Chao1 is also an estimator for species richness but more weight is given to the low abundance species^37^. Subsequently, the rarefaction curves were plotted using the data from Observed species.

### Statistical analysis

The microbial OTUs were compiled and grouped into different taxonomic levels while OTUs belonging to chloroplast, mitochondria and unassigned were excluded from the analysis^28^. All statistical analyses were performed using R version 3.5.1 and all data were tested for normality using the Shapiro–Wilk test. For the normally distributed data, analysis of variance (ANOVA) was used to test the significant differences based on forest types and depths and followed by post hoc Tukey HSD test using the ‘aov’ and ‘TukeyHSD’ functions respectively while for the non-normally distributed data the Friedman test was utilized along with pairwise Wilcox test in case of significant results using the ‘friedman.test’ and ‘pairwise.wilcox.test’ functions.

Further, non-metric multidimensional scaling (NMDS) with 999 permutations was performed using the ‘metaMDS’ function based on Bray–Curtis distant matrices. In order to determine the correlation between the peat soil chemical properties and environmental variable with the microbial community, the ‘envfit’ function was used with 999 random permutations. Lastly, permutational multivariate analysis of variance (PerMANOVA) was utilized to assess the statistical significance of microbial data in the different forest types, soil depth and months using the ‘adonis’ function. The relationship between forest types and the major prokaryotic taxa was visualized using principle component analysis (PCA).

### Data accessibility

Sequencing data were submitted to NCBI Sequence Read Archive database (http://www.ncbi.nlm.nih.gov/sra/) under the Bioproject number PRJNA588113.

## Results

### Differences in water table and soil chemical properties

During this study the Maludam National Park has a mean annual precipitation of 3182 mm and mean annual temperature of 27.2 °C from 2017 to 2018. The water tables were significantly different (*p* < 0.05) only for ABg as the water level is constantly above ground surface (Supplementary Table S2). As for MPS and ABt, the water tables fluctuated in the top 0–20 cm soil layer throughout the sampling period, keeping the 30–50 cm soil layer constantly submerged under water. Submerged soil layers (30–50 cm) were observed to be more acidic than soil layers that was not submerged (0–20 cm) at MPS and ABt. Peat chemical properties that showed significant differences (*P* < 0.05) were pH and PSI. Higher pH (above pH 4.1) and lower PSI (below PSI 4.9) were observed for ABt and ABg. Higher PSI values in MPS (Table 1) indicated that it has higher degree of humification, whereas lower PSI values in ABt and ABg (Table 1) showed lower degree of humification. The LOI analysis showed that the organic matter contents in the soil samples were more than 98%, which were of normal range for organic soil. However, there were no significant differences observed for total C, total N and C/N ratio. The humification level of the soil from the three peat forest types were in this case, unable to be differentiated based on the C/N ratios except that it gave useful information on the carbon and nitrogen contents. Therefore, PSI may be a more reflective parameter for humification level at the three sites for this study.

**Table 1:** Peat soil physicochemical properties of MPS, ABt, and ABg at 0–20 cm and 30–50 cm soil depths.

Properties	MPS		ABt		ABg	
	0–20 cm	30–50 cm	0–20 cm	30–50 cm	0–20 cm	30–50 cm
**WT (cm)**	− 3.8 ± 3.6^a^		− 8.1 ± 4.7^a^		18.3 ± 3.8^b^	
**pH**	3.9 ± 0.0	3.8 ± 0.0	4.2 ± 0.0	4.1 ± 0.1	4.1 ± 0.0	4.1 ± 0.0
**LOI (%)**	98.5 ± 0.2^a^	99.3 ± 0.1^b^	99.4 ± 0.1^b^	99.5 ± 0.1^b^	99.4 ± 0.1^b^	99.6 ± 0.0^b^
**PSI**	21.5 ± 1.9^a^	13.5 ± 0.8^b^	2.5 ± 0.1^c^	4.9 ± 0.6^c^	1.9 ± 0.2^c^	1.9 ± 0.1^c^
**TC (%)**	53.9 ± 0.7^a^	53.9 ± 0.7^a^	54.1 ± 1.1^a^	54.1 ± 0.9^a^	54.0 ± 1.0^a^	54.5 ± 0.6^a^
**TN (%)**	1.5 ± 0.1^a^	1.7 ± 0.2^a^	1.7 ± 0.1^a^	1.7 ± 0.0^a^	1.5 ± 0.1^a^	1.5 ± 0.1^a^
**C/N ratio**	36.1 ± 3.0^a^	33.4 ± 3.6^a^	32.3 ± 2.6^a^	32.6 ± 0.8^a^	35.4 ± 1.9^a^	36.5 ± 2.3^a^

Values are mean ± standard error. Different letters in the same row indicate significant difference at the level of *P* < 0.05 based on Tukey’s HSD test.

*WT* water table with (−) values indicating depths below soil surface, *LOI* loss-on-ignition, *PSI* pyrophosphate solubility index, *TC* total carbon, *TN* total nitrogen.

### Variations in alpha diversity

The average number of high-quality reads obtained from all 24 samples was 31,138 (Supplementary Table S1). A total of 50,245 operational taxonomic units (OTU) at 97% similarity were obtained. The rarefaction curves of the Observed species across all 24 samples were tabulated (Supplementary Figure S1). The microbial alpha diversity was evaluated by the Shannon diversity index, Chao1, Observed species and PD whole tree (Table 2, Supplementary Figure S2). Statistical analyses were conducted and showed that alpha diversity indices differ significantly among forest types (*p* < 0.05) as shown primarily by the Observed species and PD whole tree. At the top layer of 0–20 cm, microbial species diversity (Observed species and PD whole tree), evenness (Shannon index) and abundance (Chao1) were highest in the following order ABt > ABg > MPS and as for the bottom layer of 30–50 cm, it was in the following order ABg > ABt > MPS except for evenness.

**Table 2:** The alpha-diversity indices of prokaryotic microbiota in the different peat swamp forest types based on soil depth.

Depth and forest types	Shannon Index^2^	Chao1^1^	Observed Species^1^	PD Whole Tree^1^
**0–20 cm**				
MPS	8.29 ± 0.11^a^	10,918.79 ± 276.90^a^	3385.8 ± 62.8^a^	143.50 ± 2.08^a^
ABt	9.01 ± 0.04^b^	12,042.47 ± 3 12.75^b^	3958.3 ± 51.2^b^	170.39 ± 2.00^b^
ABg	8.92 ± 0.01^b^	11,030.92 ± 159.09^a^	3717.2 ± 13.5^c^	161.98 ± 0.71^c^
**30–50 cm**				
MPS	8.26 ± 0.08a^b^	9234.13 ± 369.47^a^	3207.5 ± 90.7^a^	141.89 ± 2.87^a^
ABt	8.00 ± 0.09^a^	10,123.32 ± 339.99^a^	3221.1 ± 78.3^a^	147.08 ± 2.95^a^
ABg	8.59 ± 0.07^b^	11,867.66 ± 358.93^b^	3662.1 ± 81.5^b^	162.30 ± 3.03^b^

Values are mean ± standard error. Rarefied samples (22,738 sequences) were used for the analyses. Different letters in the same column indicate significant difference at the level of *P* < 0.05 based on Tukey’s HSD test (^1^) or pairwise Wilcox test (^2^).

### Differences in dominant taxonomic groups

**Figure 2:**
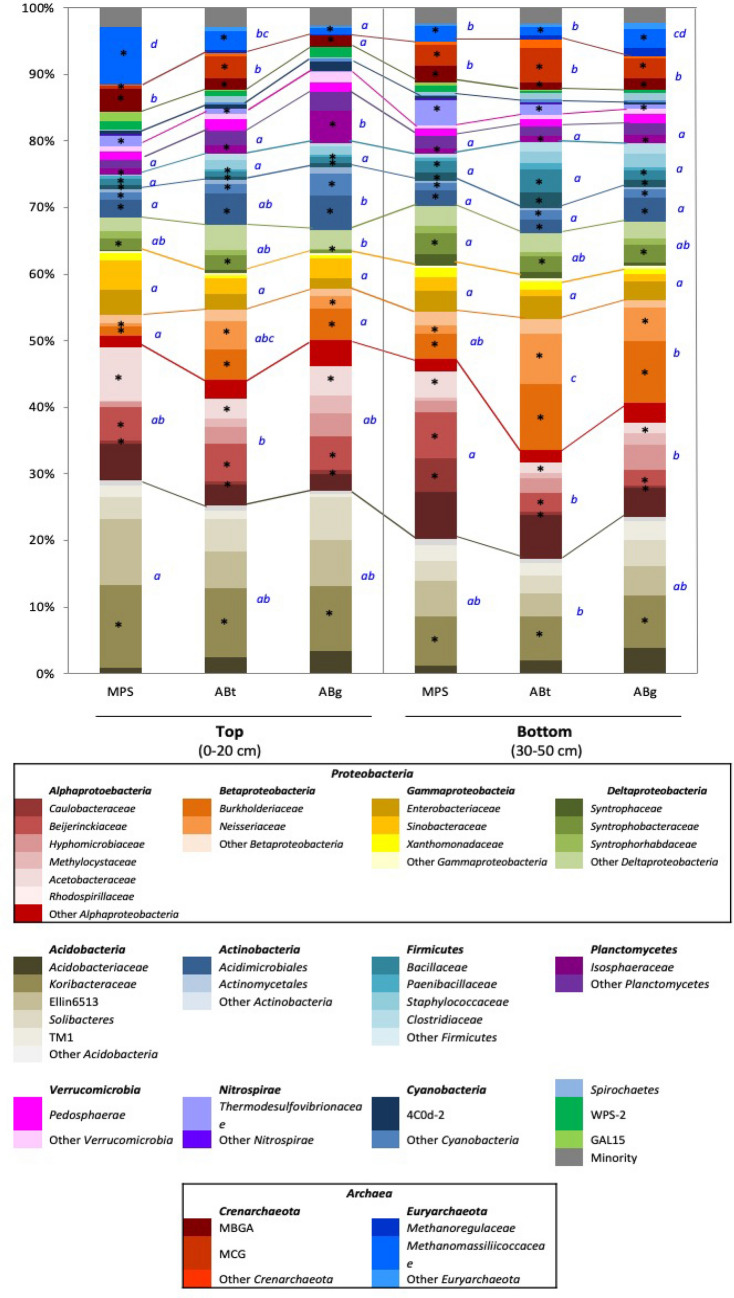
Comparison of prokaryotic microbiota composition in MPS, ABt, and ABg forest at two depths, 0–20 cm (T) and 30–50 cm (B) below the soil surface. Only those taxa with relative abundance higher than 1% were shown and each colour represents a particular taxon of prokaryotic groups. MBGA represents Marine Benthic Group A and MCG represents Miscellaneous Crenarchaeotic Group.

The average relative abundance of bacteria was 88.5% (84.4–94.3%) and the remaining percentage represented archaea (Fig. 2). There were 45 prokaryotic microbiota phyla identified in the Maludam tropical peat swamp forests with 13 dominant phyla (relative abundance higher than 1%). The most abundant phyla in all forest type are Proteobacteria and Acidobacteria taking up a combined total of an average of more than 70% of the whole prokaryotic population (Fig. 2). Sequences that were less than 1% in both forest types and depths were clustered together forming the minority phyla comprising of Parvachaeota, Armatimonadetes, Bacteroidetes, Chlamydiae, Chlorobi, Chloroflexi, Elusimicrobia, Fibrobacteres, Fusobacteria, Gemmatimonadeles, Lentispharae, Synergiseles, Tenericutes*,* AD3*,* BH180-139, BRC1, FBP, FCP0426, GNO2, NC10, NKB19, ODI, OP11, OP3, SAR406, SRB1093, SPI, TM6, TM7, TPD-58, WS4 and WWE1.

The relative abundance of the phylum Acidobacteria was the highest in the top layer of MPS (29%) and was significantly different to the percentage in ABt forest which had the lowest value especially in the bottom layer (17.3%) (Supplementary Table S3). Alphaproteobacteria, one of the classes of Proteobacteria, showed significantly higher abundance in MPS than the other two forests especially in the bottom soil layer with a percentage of 27.1%. It was observed that ABt forest possessed the highest percentage of the class Betaproteobacteria (top: 10.6%, bottom: 19.7%) followed by ABg and MPS respectively. The Gammaproteobacteria group showed no significant difference throughout the three forests. As for Deltaproteobacteria, the relative abundance of this group at the top layer was highest in ABt (7.2%) while at the bottom layer it was highest in MPS forest (8.9%). For Actinobacteria, a significant difference in percentage was observed only at the top soil layer with the highest in ABg, followed by ABt and MPS. The phylum Firmicutes has higher percentage in ABt and ABg forests especially in the deeper soil layers although there was no significant difference observed at *P* < 0.05. The phylum Planctomycetes exhibited high relative abundance in ABg forest specifically in the top layer and was significantly different from the other two forest types. The relative abundance of the phylum Verrucomicrobia was the lowest in MPS forest, intermediated in ABt forest and highest in ABg forest in both depths. Other microbial groups detected were Nitrospirae, Cyanobacteria, Spirochaetes, WPS-2 and GAL15. Crenarchaeota and Euryarchaeota were the two dominant phyla from the archaea domain. Crenarchaeota was observed to be highest in ABt forest, whereas Euryarchaeota was observed to be significantly greater in abundance at the top layer of MPS forest.

A PCA (Fig. 4) was conducted based on the relative abundance of the major bacterial and archaeal phyla. The first two axis of the PCA explained about 58.3% of the total variance, with PC1 and PC2 explained 37.5 and 20.8% of the total variance, respectively. The PCA analysis showed a pattern of distribution for prokaryotic microbiota communities from different forest types and soil depths. There were four major groups observed in the clustering of prokaryotic microbiota; (a) MPS(T), (b) MPS(B), (c) ABg(T) and (d) ABg(B), ABt(T), ABt(B). The MPS(T) soil is correlated with the relative OTU abundance of Acidobacteria, Alphaproteobacteria, Gammaproteobacteria and Euryarchaeota taxa whereas, MPS(B) was highly correlated to Deltaproteobacteria and Nitrospirae taxa. As for ABg(T), the relative abundance of the phyla Actinobacteria, Planctomycetes, Verrucomicrobia, and Cyanobacteria showed correlation. The remaining three ABt(T), ABt(B) and ABg(B) clusters were observed to be overlapped and was highly correlated to the relative abundance of Betaproteobacteria, Firmicutes and Spirochaetes.

### Association between soil and environmental parameters with prokaryotic community composition

**Figure 3:**
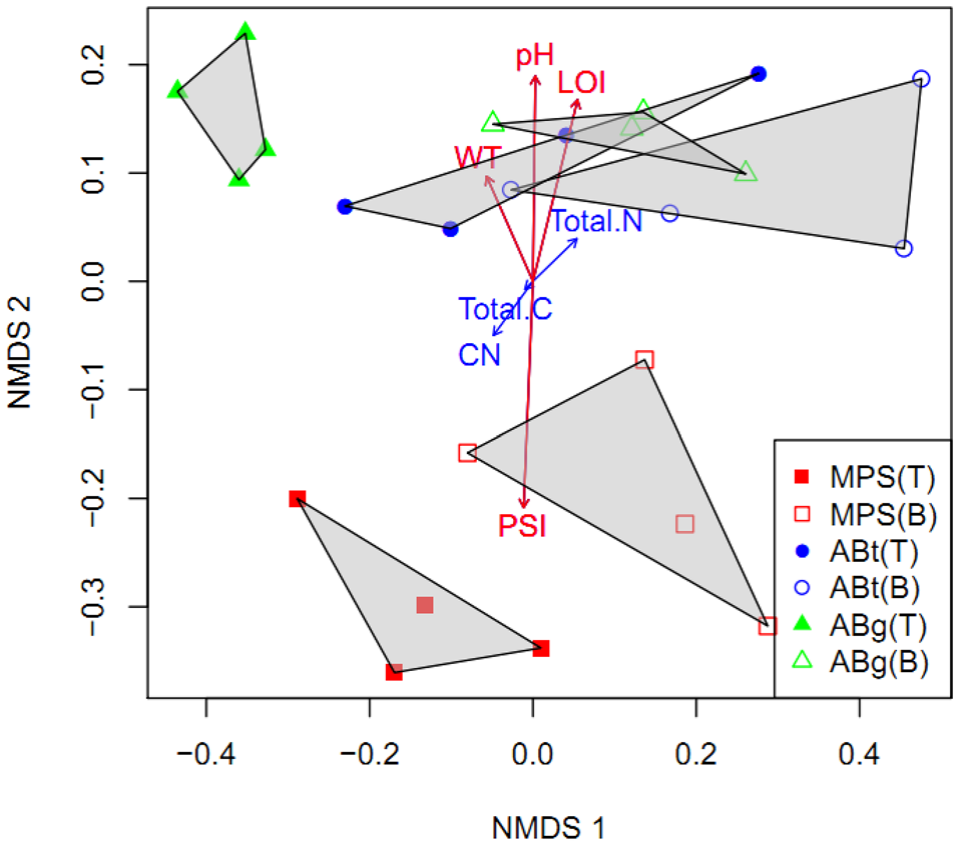
Non-metric multidimensional scaling (NMDS) analysis based on Bray–Curtis distant matrices of prokaryotic microbiota, forest types and soil depth. A vector overlay of the physicochemical variables is shown on the plot. Vectors shown in red color are the significantly correlated variables; PSI, pH, LOI and WT.

The PerMANOVA analyses indicated that forest types and soil depths significantly influenced the microbial community composition in this study (*P* < 0.01) while no significant effect of different months was detected (*P* < 0.127). Forest types explained about 24% whereas depth explained 19% of the compositional variation in the communities. Based on the NMDS ordination plot, prokaryotic community compositions were segregated by forest types and depths (Fig. 3). The plot displayed a clear separation of MPS prokaryotic community composition from that of ABt and ABg forests, with both ABt and ABg forests showed an indication of overlap. In particular, the overlap occurred between prokaryotic community from the topsoil samples of ABg with the top and bottom samples of ABt forest. In order to further investigate the relationship of the soil parameters and environmental variable on the prokaryotic community composition, vectors were fitted onto the ordination space using ‘envfit’ function. Among the six soil chemical properties and one environmental variable analyzed, four were found to be significantly correlated with the microbial community compositions (*P* < 0.05). PSI showed the highest correlation (r^2^ = 0.8674), followed by pH (r^2^ = 0.7149), LOI (r^2^ = 0.6179) and WT (r^2^ = 0.2515).

## Discussion

This study provides insights of prokaryotic community composition differences in soils from three forest types with different oligotrophic environment on a tropical peat dome in Maludam National Park, Sarawak, Malaysia: MPS (least oligotrophic), ABt (moderately oligotrophic) and ABg (most oligotrophic) forests. The oligotrophic level of the three forests types were also described by Monda et al.^30^, where trees were observed to develop higher wood strength, stem thickness but hollow stem in moderately oligotrophic conditions of ABt forest, while trees invest more in stem thickness than wood strength in the severely oligotrophic ABg forest. Previous work conducted using next-generation sequencing in the tropical peat swamp forest of Thailand^25^, Brunei^27^ and Peninsular Malaysia^28^ generalize tropical peat swamp forest to only one type. However, the pioneering work by Anderson^15^ previously described that there are six different forest types of the tropical peat swamp forest based on the forest vegetation in Borneo. The distinct chrono sequence of forest types across a tropical peat dome reflects the evident difference in characteristics of the underlying peat soils. It is documented that peat decomposability rate for the three forest types is in the order of ABg > ABt > MPS^19^ and peat accumulation rate also corresponds in the same order^29^, which translates into the decomposition level from highest to lowest in the reverse order of MPS > ABt > ABg. It was hypothesized that microbial community composition will be reflected in concordance with the peat soil decomposition level. However, the abundance and diversity of the prokaryotic microbiota were found to be in the order of ABt > ABg > MPS in the 0–20 cm soil layer and the order ABg > ABt > MPS in the 30–50 cm soil layer.

### Soil chemical properties affect composition of prokaryotic microbiota

This study found that the soil chemical properties of soil had significant influence on the composition of prokaryotic microbiota in the different peat forests type. The prokaryotic community compositions in ABt and ABg were influenced by the less acidic condition and higher organic matter content (Table 1, Fig. 2). This is in accordance with the bigger soil aggregates in ABt and ABg, containing more fresh organic materials. The organic matter from plant origin consists of varying amounts of organic compounds, which are differentiated according to molecular size, solubility and primary constituents. These organic compounds in the normal course of decomposition will undergo a sequential decay process and this may be related to the plant fiber structure as microorganisms may have access to those that are located on the more available outer structure^42^. In the initial stage of organic matter decomposition, materials particularly sugars, low-molecular-weight phenolics and some nutrients are readily lost through dissolution and leaching. In addition to that, opportunistic microorganisms will be rapidly growing and feeding on the remaining simpler materials^43^. In principle, the next targeted organic compound is the hemicelluloses particularly arabinan based and followed by galactans, mannans and xylans successively. The onset of cellulose degradation is expected to be the same as mannans and lignin degradation comes last due to the slow growing lignin-degrading microorganisms mainly the white-rot, soft-rot, brown-rot fungi^44^ and the more recent reports of lignin-degrading bacteria such as Actinomycetes, Alphaproteobacteria and Gammaproteobacteria^45,46^. However, there are findings reporting that there is also a possibility of the onset of lignin degradation immediately after litter fall^47^. In the complex forest ecosystem, individual processes may be dominating but all processes may occur continually throughout the decay continuum. These processes are dominated by decomposer microorganisms through the production of extracellular hydrolytic enzymes that are either bound onto the outside of the cell or released to its surrounding environment^43^. Therefore, the higher content of organic matter in ABt and ABg could be the main reason for the higher prokaryotic microbiota abundance as it attracts decomposer microorganisms especially those that degrade hemicellulose and cellulose.

As organic matter decomposes, organic carbon and nutrients are either absorbed by plants or transferred to the soil organic matter (SOM), which can be categorized into three major pools; dissolved organic matter (DOM), particulate organic matter (POM) and humus. For the DOM and POM pools, they will be continually being decomposed by microorganisms whereas humus is in the most decomposed form, is more resistant to further microbial decomposition^48^. The stable humus contains the insoluble fraction known as humin and the soluble fraction consisting of humic and fulvic acid. It is generally characterized as dark brown to black pigments found in soil and is rich in oxygen-containing functional groups, notably carboxyl, phenolic, enolic, alcoholic and carbonyl of quinones^2^. It is observed that the humification level at MPS was higher as reflected in the ten-fold PSI value in comparison to the lowest level in ABg (Table 1). Thus the lower pH reading in this study could be due to the higher humus component. Therefore, the prokaryotic community abundance in MPS was distinctly different from ABt and ABg (Fig. 2) and driven by these two factors; higher humification level and lower pH. Higher humification level at MPS could be due to localized source or washed-down from the upstream ABt and ABg forests. This information could be used to better understand the biogeochemical mechanisms that regulate soil microbial community composition and maintaining its functionality is crucial for the improvement of peatland management.

### Higher prokaryotic diversity and abundance in the more oligotrophic forests

The results of this study indicated higher prokaryotic microbiota diversity and abundance in the more oligotrophic peat swamp forest in ABt(T) and ABg(B). Despite the fact that ABg(B) soil layer was constantly submerged in water under anoxic condition (Supplementary Table S2), a higher prokaryotic diversity and abundance was observed. One common understanding is that the soil microbial activities in tropical peatlands are inhibited due to the acidic waterlogged condition caused by the fluctuating groundwater that divides the soil into two distinct layers; the oxic-acrotelm layer and the permanently water saturated anoxic-catotelm layer^49^. However, it could be that tropical peatland ecosystems are dominated by anaerobes and fermenters such as Acidobacteria, Proteobacteria, Actinobacteria and Firmicutes that covers 80–90% of the total prokaryotic abundance. Members of the phyla Actinobacteria and Proteobacteria have been associated to anaerobic respiration in peat^50^. In bottom layer of soil located below the water table and is constantly anoxic, is usually dominated by anaerobic degradation that takes place at lower decomposition rate than aerobic degradation. Under the anaerobic conditions, organic matter is degraded to methane (CH_4_) and carbon dioxide (CO_2_) as the end products^51^.

The more oligotrophic forests ABt and ABg have higher abundance and diversity in prokaryotic microbiota and this could be due to bigger soil aggregates observed in the two forest types. The soil macroaggregates contain more fresh organic materials of plant deposits in the form of leaf litter, wood residue and root mass that are more susceptible to microbial decomposition. With time, these macroaggregates will be broken down to form microaggregates. Microaggregates are formed from primary particles from plant and microbial debris bound together by humic materials and polysaccharide polymers that protects soil organic matter (SOM) against decomposition. The aggregate structure has an important role in the accessibility of SOM for microbial decomposition^52^, which is also reflected by higher CO_2_ emission rate from peat macroaggregate in an incubation study^53^. The other reason for the higher abundance of prokaryotic microbiota in ABt and ABg forests could be that the aromatics by-products from the breakdown of fresh organic materials especially labile carbohydrates are washed-off due to the higher water flow through the area. These aromatic by-products that are known to actively inhibit anaerobic decomposition^13^ are not accumulated and therefore allow higher activity of anaerobic decomposition in ABt and ABg forests. This is supported by the higher CH_4_ flux from ABt and ABg forests in comparison to MPS from the preliminary soil respiration chamber experiment (unpublished) (Supplementary Figure S3). Another rationale for the lower aromatic by-product inhibition in ABt and ABg is the existence of microbes that can catabolize aromatic compounds in these two forest types. In this study, it was found out that *Burkholderia oxyphila* from the class of Betaproteobacteria was higher in abundance in ABt and ABg forests than in MPS which reinforced this fact. *Burkholderia oxyphila* has been reported to be able to catabolize various aromatic compounds such as catechin, vanilic acid, protocatechuic acid, p-hydroxybenzoic acid, 4-hydroxy-3-methoxycinnamic and trans-pcoumaric acid in a forest soil in Japan^54^ (Table 3). In 2014, all *Bukholderia* genus which are primarily environmental samples were transferred to a new proposed genus; *Parakburkholderia* gen. nov. Thus, the name of *Burkholderia oxyphila* was amended to *Paraburkholderia oxyphila*^55^. Additionally, ABt forest was found to be abundant with *Aquitalea magnusonii* which has been reported to degrade aromatic compounds as well^56^. There are possibilities of other microbes that has the same ability that was not captured in the data of this study. These may explain the low activity of anaerobic decomposition in MPS even though considerable percentage of methanogens mainly from the phylum Euryarchaeota resided in this forest as the aromatic by-products impeded the anaerobic decomposition process thus less CH_4_ gas. Moreover, *Methylovirgula ligni* was found to be abundant in MPS which is from the methanotroph family of Beijerinckiaceae that consume CH_4_ contributing to the low CH_4_ gas emissions in MPS forest^57^.

**Table 3:** Compilation of major operational taxonomic units (OTUs) from each family, their associated identity and potential function/role in carbon cycling. No information (ND) was found for most of the OTUs that are associated with uncultured bacterium/archaeon.

Phylum/class	Family	OTU	Associated species	Accession no.	Function/role
**Acidobacteria**	Koribacteraceae	23,592	Uncultured bacterium	MH532163.1	ND
		38,977	Uncultured bacterium	FJ166836.1	ND
**Alphaproteobacteria**	Beijerinckiaceae	30,093	*Methylovirgula ligni*	CP025086.1	(a) Utilize reduced carbon substrates with no carbon–carbon bonds as sole source of carbon and energy (b) Major carbon source is methanol, which is produced from decomposition of fallen woody materials and plant debris (c) Capable of atmospheric nitrogen fixation (Lidstrom^91^, Vorob’ev et al.^92^)
	Hyphomicrobiaceae	3769	*Rhodoplanes oryzae*	NR_134156.2	(a) Can undergo photoorganoheterotrophy or chemoorganoheterotrophy (Srinivas et al.^93^)
	Rhodospirillaceae	17,532	Uncultured bacterium	MF439272.1	(a) Can undergo photoorganoheterotrophy or chemoorganoheterotrophy (b) Photoassimilate simple organic compounds under anaerobic conditions (Biebl and Pfennig^63^, Baldani et al.^62^)
		23,729	Uncultured bacterium	HQ629097.1	
		14,249	Uncultured bacterium	JQ384202.2	
	Caulobacteraceae				(a) Ability in degrading hemicellulose, cellulose and lignin (Wilhelm et al.^64^)
**Betaproteobacteria**	Burkholderiaceae	18,034	*Paraburkholderia metalliresistens*	MN661271.1	(a) Assimilates 1-aminocyclopropane-1-carboxylate (ACC), which indirectly promotes plant growth (b) Resistant to metals and has phosphate-solubilizing ability (Guo et al.^94^)
		28,966	*Paraburkholderia* *oxyphila *(*Burkholderia oxyphila*)	NR_112887.1	(a) Able to transforms catechin, a component of tannins into taxifolin through a two-step oxidation process under the acidic conditions of a forest soil in Japan (b) Catabolizes various aromatic compounds such as catechin, vanilic acid, protocatechuic acid, p-hydroxybenzoic acid, 4-hydroxy-3-methoxycinnamic and trans-pcoumaric acid (Otsuka et al.^54^, Sawana et al.^55^)
		40,794	*Burkholderia arboris*	NR_042634.1	(a)Assimilates L-arabinose, D-mannitol, N-acetylglucosamine, maltose, caprate and phenylacetate (b)Produces pyocelin, a bacterial siderophore with bioactivities from generation of reactive oxygen species. These pyocelin damage plant tissues and is linked to pine wilt disease (Vanlaere et al.^95^, Mannaa et al.^96^)
	Neisseriaceae	21,128	*Aquitalea pelogenes*	MN42817.1	(a)Most active cellulose degrader involved in rapid plant litter degradation (b) Produces glycoside hydrolases that are involved in the complete enzymatic hydrolysis of cellulose (c) Have significantly higher β-d-glucosidase and cellobiohydrolase activity (Woo et al.^68^, Sedláček et al.^97^)
		37,359	*Aquitalea magnusonii*	MN709239.1	(a) Possesses genes for metabolisms of various organic acids, protein amino acids, carbohydrates and aromatic compounds (b)Plant growth-promoting characteristic with the genes for auxin biosynthesis and phosphorus supply (Lau et al.^98^, Ishikawa et al.^56^)
		29,610	Neisseriaceae bacterium	KM187040.1	ND
**Deltaproteobacteria**	Syntrophobacteraceae	47,345	Uncultured bacterium	AB364730.1	ND
		18,121	Uncultured bacterium	AB364730.1	ND
		47,874	Uncultured bacterium	LK024886.2	ND
**Planctomycetes**	Isosphaeraceae	53,553	Uncultured bacterium	MH528390.1	ND
		3315	Uncultured bacterium	KX823823.1	ND
**Actinobacteria**	Actinomycetales	23,507	Uncultured bacterium	LR589816.1	ND
		55,000	*Micrococcus* sp.	LC484762.1	(a) Produces cellulase and xylanase for lignocellulose degradation (b) Plant growth promoting ability (Dastager et al.^99^, Mmango-Kaseke et al.^100^)
	Acidimicrobiales	47,380	Uncultured bacterium	HG324890.1	ND
**Firmicutes**	Bacillaceae	7818	*Bacillus aerius*	NR_118439.1	(a) Produces endoglucanase that are important for the initiation of cellulose hydrolysis (Oke et al.^101^)
	Paenibacillaceae	46,383	*Paenibacillus tyrfis*	MN428219.1	(a) Produces active antimicrobial metabolites, plant growth promoting hormones and insecticides (Aw et al.^102^, Haruna et al.^103^)
**Nitrospirae**	Thermodesulfovibrionaceae	46,238	Uncultured bacterium	AB364847.1	ND
		12,777	Uncultured bacterium	JQ801065.1	ND
		12,230	Uncultured bacterium	KC161617.1	ND
**Crenarchaeota**	MBGA	27,465	Uncultured soil archaeon	HQ614127.1	ND
		6922	Uncultured bacterium	MH532224.1	ND
		25,348	Uncultured bacterium	MH532218.1	ND
	MCG	55,141	Uncultured soil archaeon	MK527582.1	ND
		50,789	Uncultured soil archaeon	KF640358.1	ND
		23,899	Uncultured soil archaeon	AB600448.1	ND
**Euryarchaeota**	Methanomassillicoccaceae	15,675	Uncultured soil archaeon	AB364937.1	(a) Available in pure culture. Uses H_2_ as electron donor but is not able to reduce CO_2_ to CH_4_ (b) Ability to reduce methanol to CH_4_ and also contain complete sets of genes for the degradation of methylamines to CH_4_ and NH_3_ (Iino et al.^104^, Borrel et al.^90^, Nobu et al.^105^)

Tropical peat swamp forests although characterized as waterlogged, nutrient-poor, anaerobic and highly acidic in nature, these ecosystems could still support complex microbial communities as any other terrestrial ecosystem. Tripathi et al.^27^ also reported that tropical peat swamp forest could be as microbiologically diverse as a tropical mixed dipterocarp forest. However, dominant microbes may be novel or inadequately characterized due to limitation in isolating pure cultures. One of such examples is the comparatively new Acidobacteria phylum found in this study as they are dominated by Koribacteraceae, Solibacteres and Ellin6513. Koribacteraceae and Solibacteres are previously grouped as the bacterium Ellin and have only been recently characterized^58^. *Acidobacteria capsulatum* was the first described member of Acidobacteria only in the 1990’s^59^. Therefore, the unknown and the uncultured microbes could possibly be the dominant group that is greatly under-estimated in their diversity and abundance in tropical peatland ecosystems.

### Major prokaryotic taxa and their functions in carbon cycling

As has been shown in this study that the microbial communities in tropical peat swamp forests could be as diverse and abundant as any other tropical forests, except that these ecosystems are dominated by mostly the lesser known microbes. Their functional roles especially in relation to C cycling is important for the understanding of tropical peatland ecosystems as terrestrial carbon stores. The basis to understand these specific roles is, all microbes transfer carbon between environmental compartments to achieve one fundamental goal, that is to survive through reproduction. They live by using different organic and inorganic forms of carbon as carbon and energy sources^60^. Therefore, it is essential to elucidate the C utilization preference by the major prokaryotic taxa found in the tropical peat swamp forests.

Acidobacteria is a relatively new phylum found to be abundant and diverse across various ecosystems especially in soil. However, due to the difficulty in cultivation, relatively little information is available to describe the ecological functions of the members of this phylum^58^. In this study, Acidobacteria covers 20–30% of the total relative abundance of prokaryotic microbiota in each soil. The major members of this phylum were found to be Koribacteraceae, Solibacteres and Ellin6513. However, *Koribacter versatilis* and *Solibacter usitatus,* previously known as Ellin6513 and Ellin6076 respectively, were the only two isolates representatives of Koribacteriaceae and Solibacteres. They are not fully characterized and do not possess valid taxonomical name to date^58^. Nevertheless, the abundant presence of this phylum in soils may suggest that these microbes play an important role in biogeochemical processes^61^, specifically in plant cell wall degradation since it has been demonstrated to degrade xylan^58^.

**Figure 4:**
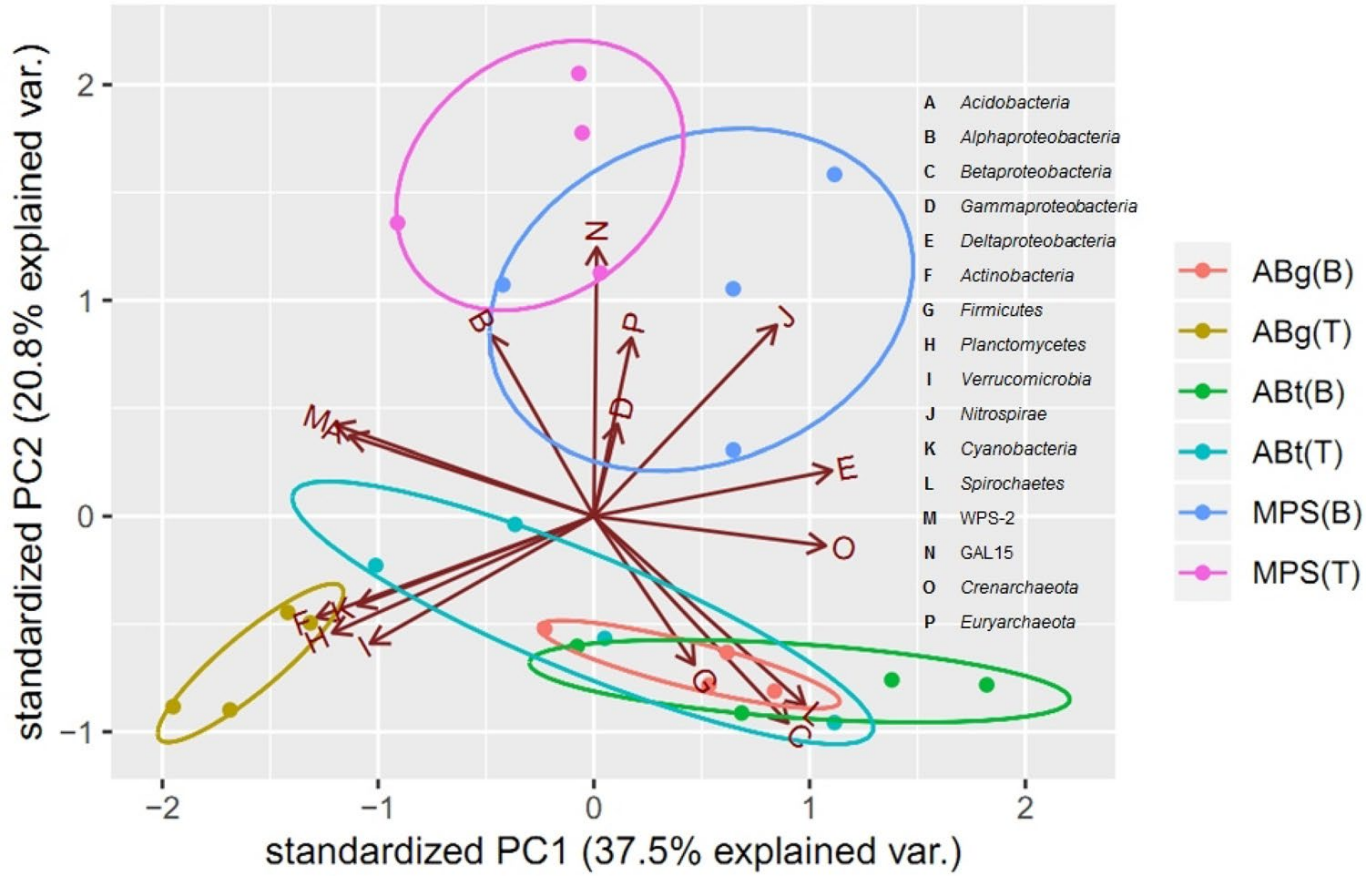
Principal component analysis (PCA) of forest types and the major prokaryotic taxa in Maludam National Park. Scatter plot shows principle component 1 (PC1) versus principle component 2 (PC2). Percentages shown are percentages of variation explained by components.

**Figure 5:**
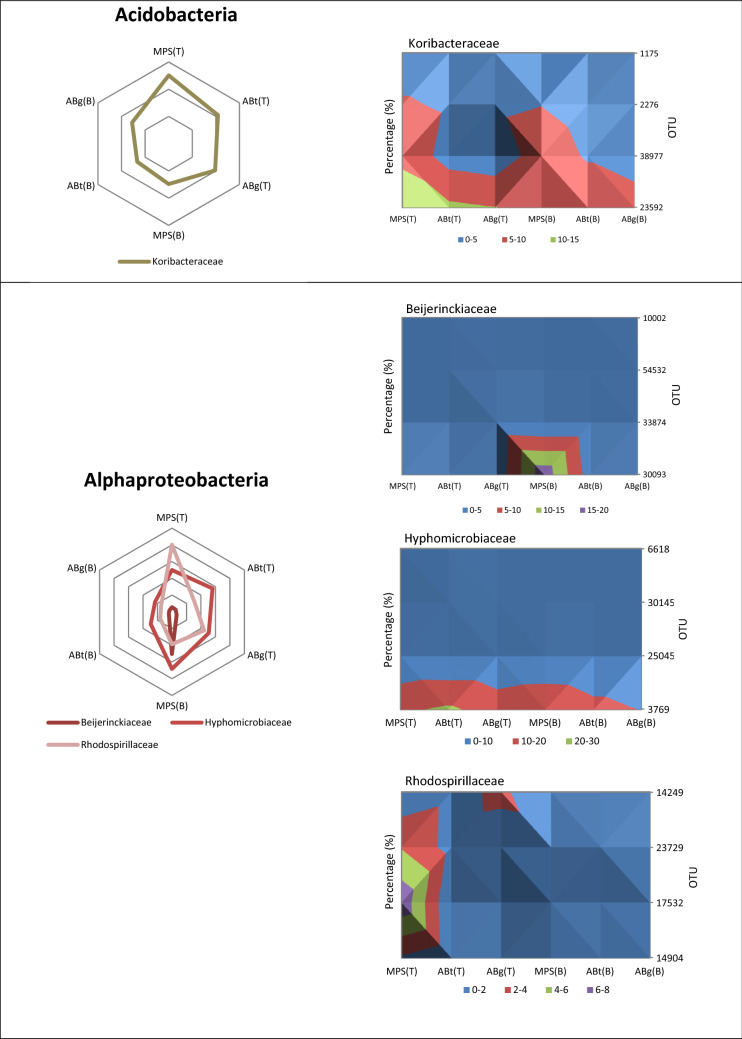

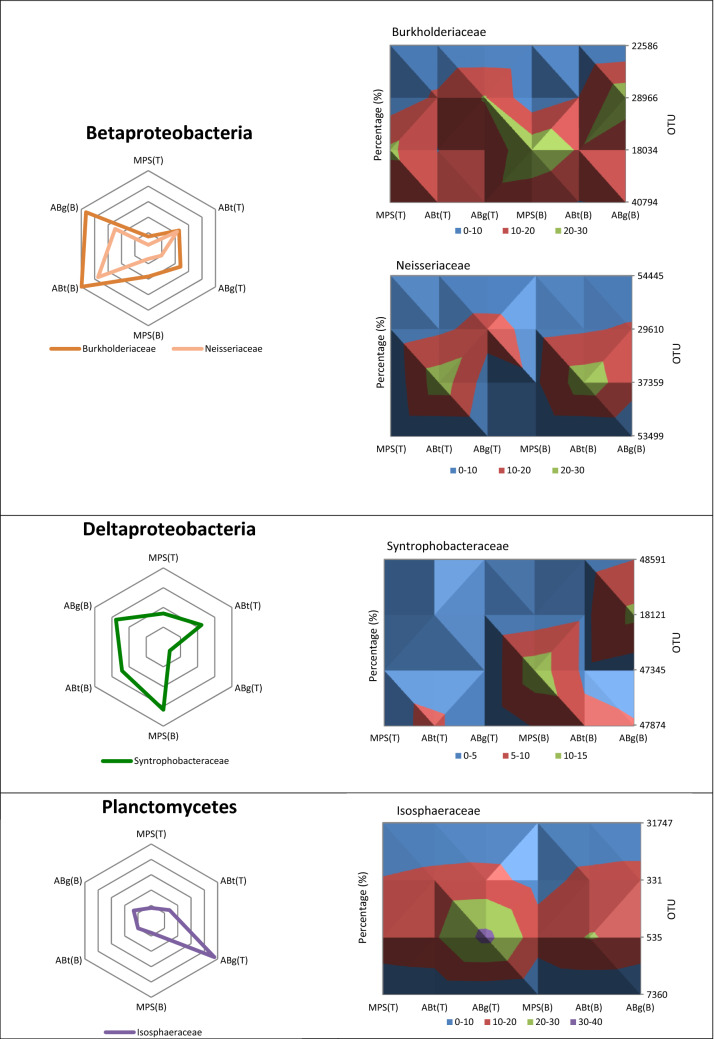

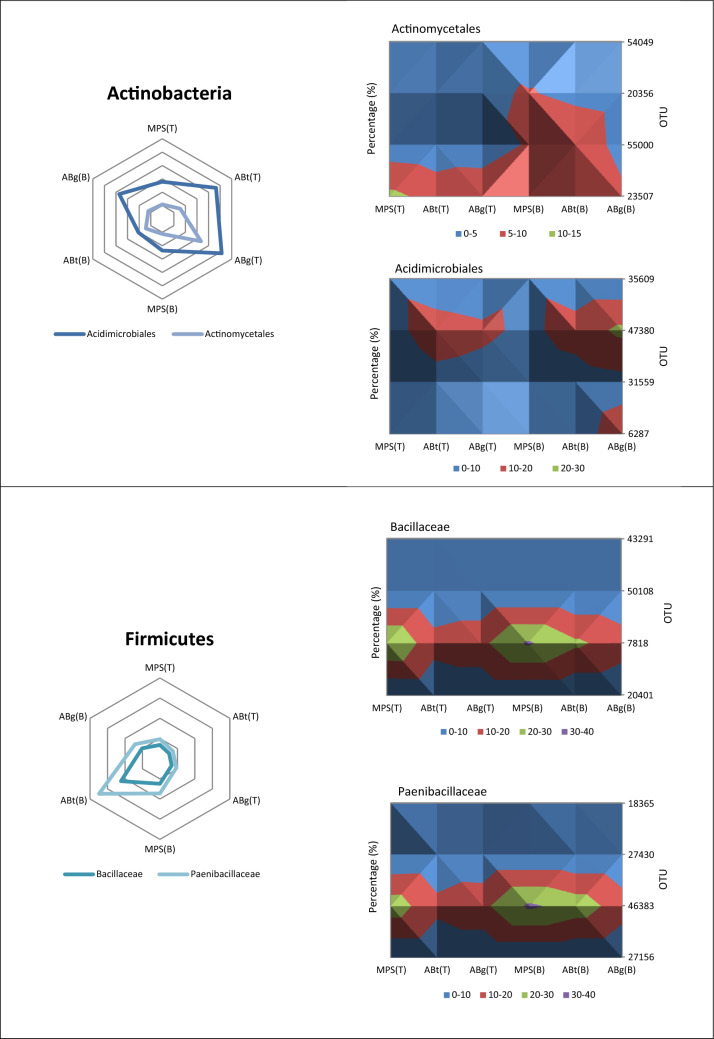

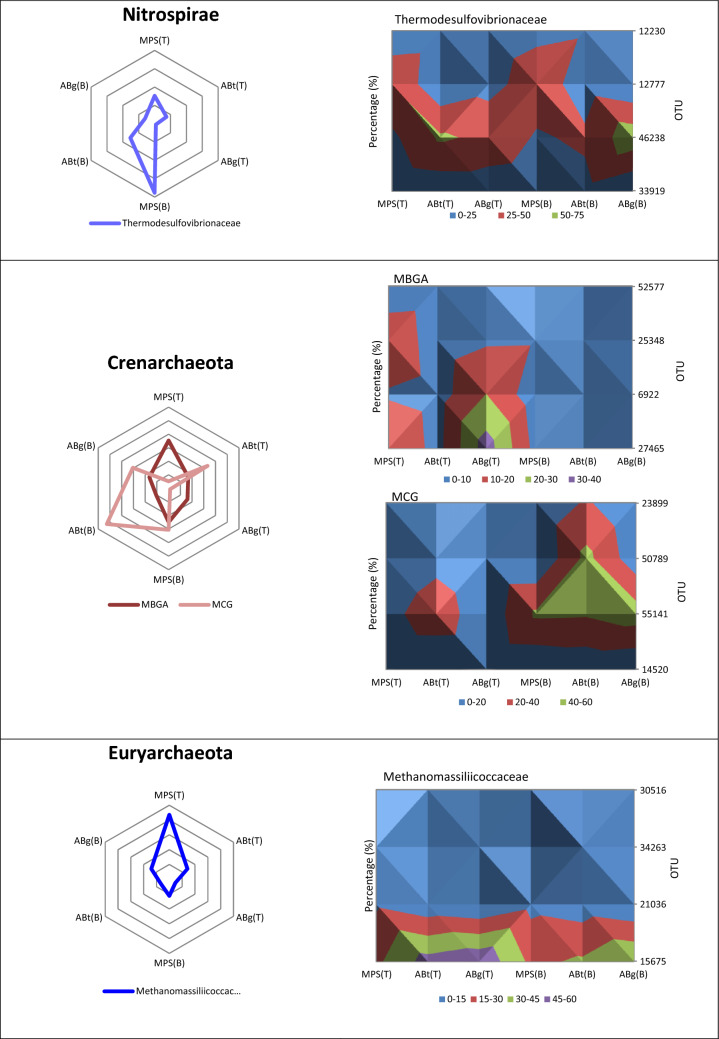
Distribution of selected prokaryotic community phyla and major operational taxonomic units (OTUs) in MPS, ABt and ABg forests. Percentage of the major OTU is calculated based on the relative abundance in their respective phylum.

The relative abundance of Alphaproteobacteria was found to be correlated to MPS forests (Figs. 4 and 5). The three major families in the Alphaproteobacteria class found were Rhodospirillaceae, Caulobacteraceae and Hyphomicrobiaceae (Fig. 2). Rhodospirillaceae is known as the purple non-sulphur bacteria and can grow photoheterotrophically under anoxic conditions in the light and chemoheterotrophically in the dark^62^. They are known to be able to photo assimilate simple organic compounds under anaerobic conditions^63^. Caulobacteraceae is reported to have the ability in degrading hemicellulose, cellulose and lignin in coniferous forest soil^64^. Hyphomicrobiaceae thrives only in the presence of low concentrations of suitable carbon sources and unable to grow in rich media. The aerobic Hyphomicrobiaceae are chemoheterotrophs whereas anaerobic Hyphomicrobiaceae grow by denitrification or mixed acid fermentation^65^.

In this study, the relative abundance of Betaproteobacteria was found to be significantly correlated to the bottom soil layers (30–50 cm) of both ABt and ABg forests (Figs. 4 and 5). The two major family of Betaproteobacteria found in this study were Burkholderiaceae and Neisseriaceae. Genus *Burkholderia* are known for their ability to degrade recalcitrant carbohydrates (mannitol, cellobiose, xylose and N-acetyl-d-glucosamine) and aromatic compounds such as L-phenylalanine^66,67^. Thus, genus *Burkholderia* could contribute to the turnover of plant derived organic carbon. As for Neisseriaceae, the genus *Aquitalea* is known to have the ability to produce hydrolytic enzymes that degrade cellulose^68^.

As for Gammaproteobacteria, the two major families are the Enterobacteriaceae and Solimonadaceae (previously named Sinobacteraceae). Many species under the Enterobacteriaceae family can exist as free-living in diverse ecological niches from terrestrial to aquatic environments, while some are only associated to animals, plants and insects^69^. They are known to be involved in decomposition of monosaccharides in aerated forest^70^. As for Solimonadaceae family, it is widely described to have the ability to decompose chemical pollutants such as atrazine, chlorinated hydrocarbons and hexane^71^.

In this study, class Deltaproteobacteria was found to be associated to the bottom soil layers (30–50 cm) at all three forest types (Fig. 5). The major family found for the class Deltaproteobacteria was Syntrophobacteraceae. Syntrophobacteraceae (*Syntrophobacter* spp. and unclassified Syntrophobacteraceae) are known to oxidize organic matter incompletely and produce acetate as the end-product. Acetate is known to be the most abundant intermediate during anaerobic decomposition of organic matter and is the most important substrate for methanogens for production of CH_4_ in anoxic environments^72^. The major family in the Nitrospirae phylum found at this soil layer is the Thermodesulfovibrionaceae, which is reported to contribute to the degradation of organic compounds and indirectly to the production of methane in anaerobic digesters^73^.

Actinobacteria was found to be significantly linked to the top soil layers (0–20 cm) in both ABt and ABg forests (Figs. 4 and 5). Actinobacteria are known to produce a range of extracellular hydrolytic enzymes to degrade plant and animal polymers including lignin, cellulose, chitin and other organic compounds^74^. In peatlands, they are reported to decompose lignocellulose by producing various enzymes such as phenol oxidase and they are also known to release dissolved organic carbon (DOC) in the form of soluble polyphenolics as end-products^75^.

Family Bacillaceae of the phylum Firmicutes is reported to be responsible for the degradation of plant polysaccharides and cellulose^76,77^. Clostridiaceae is another family under the phylum Firmicutes which is known for their fermentation capacity in acidic peat and are prominent cellobiose consumer^78^. In another report, they are known to depolymerize starch, xylan and cellulose^79^.

Planctomycetes was observed to be correlated to the top soil layer (0–20 cm) in ABg forest (Fig. 4). Planctomycetes are anaerobic autotrophs and possess genes encoding enzymes that are found only in methane-generating archaea and methane-oxidizing group of Proteobacteria. Another distinct feature of Planctomycetes is it can oxidize ammonia to dinitrogen without oxygen^80^. The major group observed in this study was Isosphaeraceae. They are known to be slow-acting decomposers of plant-derived organic matter^81^.

The Crenarchaeota phylum is said to be highly prevalent in many marine and soil systems and are more predominant over Euryarchaeota in pasture soils^82^. The result of this study also corresponds to the same finding. Crenarchaeota are known to be light-independent carbon autotrophs or simply known as the ability for carbon fixation by using CO_2_ from the atmosphere as carbon source^83,84^. The ability of using CO_2_ is different from photosynthesis as no archaea is known to carry out photosynthesis. Aside being a predominant disease-causing agent, the phylum Spirochaetes has been reported in various environments including soil but it is unclear whether they involve in C cycle process^85,86^.

In the case of Euryarchaeota, this study found that the relative abundance is highly associated to MPS(T) (Figs. 4 and 5) and this archaeal phylum is known to consist of many methanogens restricted to mostly anoxic environments^87^. Euryarchaeota facilitate anaerobic conversion of hydrogen, formate, acetate, methyl compounds and simple alcohols to CH_4_ and CO_2_ in many anaerobic ecosystems^88^. Methanomassiliicoccales is a Euryarchaeota previously known as Methanoplasmatales, which has now been validated by the International Committee on Systematics of Prokaryotes and generally accepted^89^. Methanomassiliicoccales was differentiated from the other methanogens due to its ability to grow on hydrogen (H_2_) + methyl-compounds, and therefore they are H_2_-dependent in metabolizing methyl into CH_4_^90^.

## Conclusion

Our result shows the microbial community composition was different between forest types and soil depths. Factors such as high humification level and low pH contributed to the difference in microbial community diversity and abundance in MPS forest while the more oligotrophic ABt and ABg forests were due to the less decomposed form of organic material. The comparatively fresher organic materials are more susceptible to microbial decomposition particularly anaerobic decomposition. The aromatic by-products from any decomposition processes at ABt and ABg could be lower because they are being washed-down to MPS located at a lower elevation. Also, bacteria such as *Aquitalea magnusonii* and *Paraburkholderia oxyphilla* that can catabolize aromatics compounds were found to be higher in abundance at ABt and ABg thus contributing to the low amounts of aromatic compounds. In MPS, *Methylovirgula ligni* was abundant in this forest type that could explain the low emission of CH_4_ gas since they consume methane gas. Therefore, anaerobic degradation is less inhibited at ABt and ABg compared to MPS forest as reflected in the higher CH_4_ fluxes from these forest types. Overall, the findings of this study uncover insight into the factors that influences the microbial community composition of these tropical peat swamp soil and could lay out the foundation for future study in regards to the underlying mechanisms of environmental-driven alterations in soil microbial communities and its potential implications on biogeochemical processes.

## Supplementary Information

Supplementary Information.
