# Supplementary material for: Linking prokaryotic community composition to carbon biogeochemical cycling across a tropical peat dome in Sarawak, Malaysia
Source: Sci Rep. 2021 Mar 19;11:6416. doi: 10.1038/s41598-021-81865-6 (PMC7979770; doi:10.1038/s41598-021-81865-6)
Supplement: Supplementary file 1 — Supplementary Information. [file 41598_2021_81865_MOESM1_ESM.pdf]

Supplementary Information

Linking Prokaryotic Community Composition to Carbon Biogeochemical Cycling across a Tropical Peat Dome in Sarawak, Malaysia

Simon Peter Dom[1,2], Makoto Ikenaga[3], Sharon Yu Ling Lau[1*], Son Radu[2], Frazer Midot[1], Mui Lan Yap[1], Mei-Yee Chin[1], Mei Lieng Lo[1], Mui Sie Jee[1], Nagamitsu Maie[4] and Lulie Melling[1]

[1]Sarawak Tropical Peat Research Institute, Lot 6035, Kuching-Samarahan Expressway, Kota Samarahan, 94300, Sarawak, Malaysia

[2]Department of Food Science, Faculty of Food Science and Technology, Universiti Putra Malaysia, Serdang, 43400, Selangor, Malaysia

[3]Research Field in Agriculture, Agriculture Fisheries and Veterinary Medicine Area, Kagoshima University, 1-21-24, Korimoto, 890-0065, Kagoshima, Japan

[4]School of Veterinary Medicine, Kitasato University, Towada, Aomori 034-8628, Japan

***Correspondence:** Sharon Yu Ling Lau, lauyuling@gmail.com

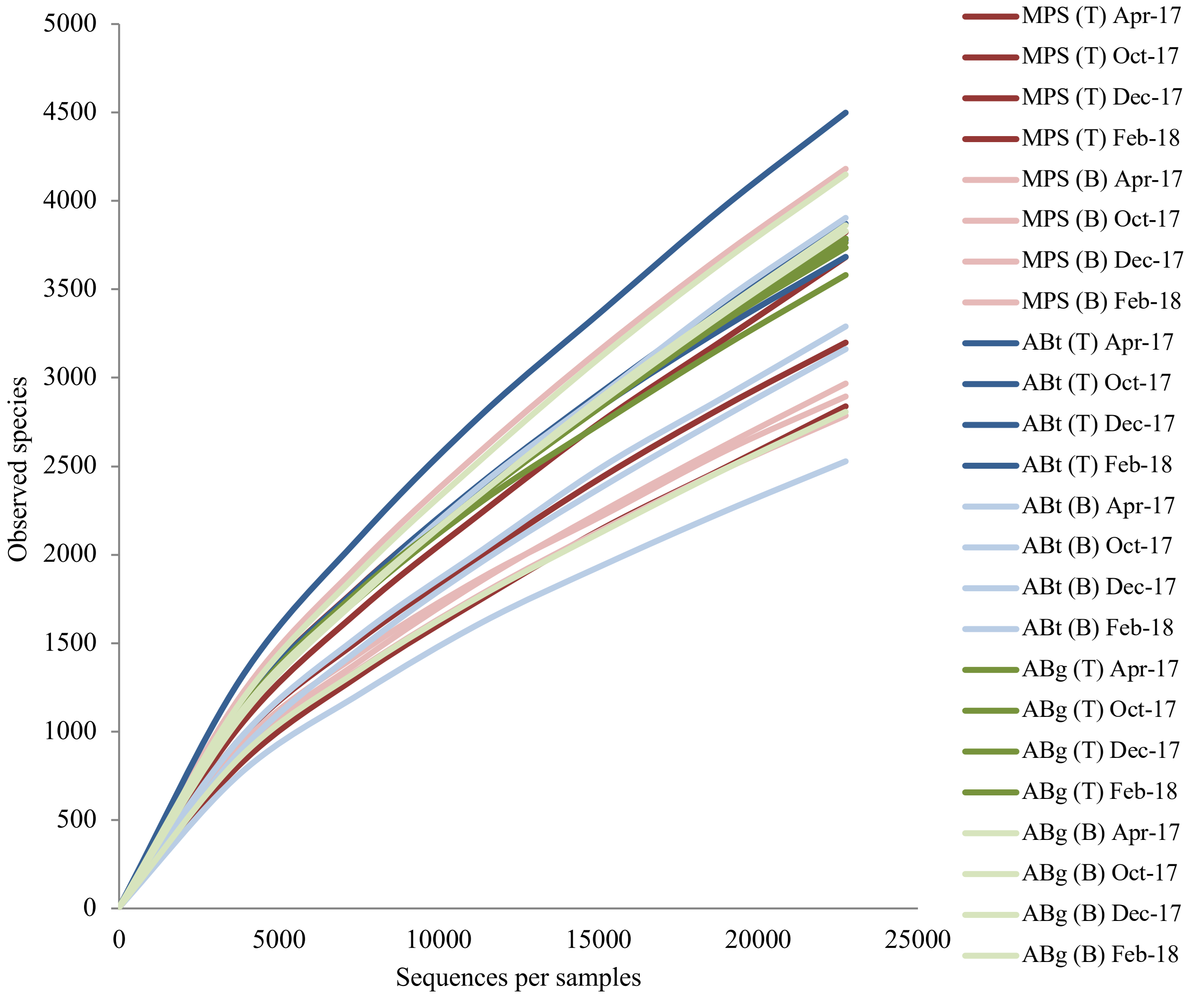

Figure S1 Rarefaction curves based on observed species of different forest types (MPS, ABt, and ABg) in two depths (T: 0-20 cm beneath the soil and B: 30-50 cm beneath soil).

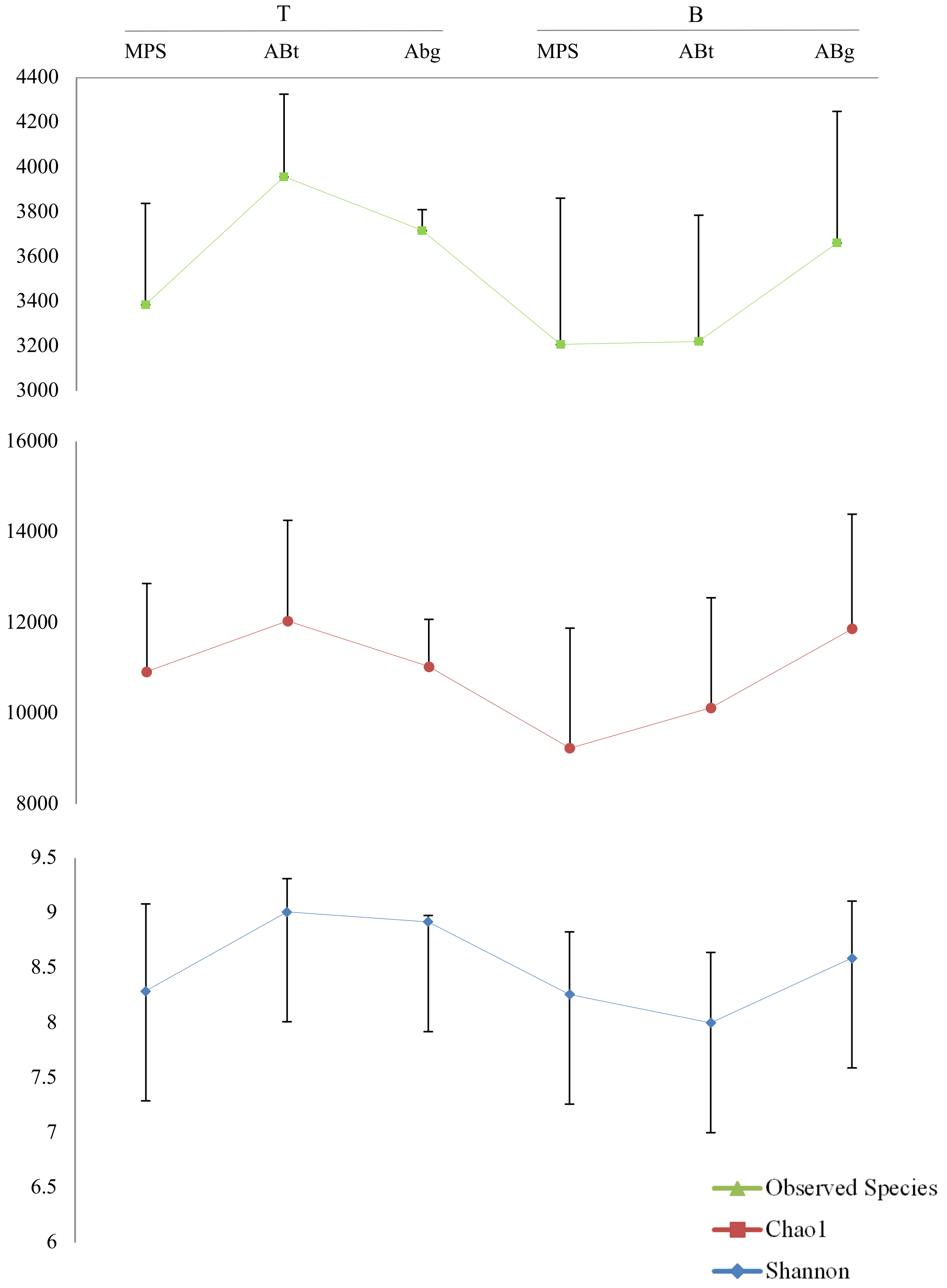

Figure S2 Microbial alpha diversity indices (Shannon, Observed species and Chao1) of each forest types (MPS, ABt, and ABg) at two depths (T: 0-20 cm beneath the soil and B: 30-50 cm beneath soil). The figure shows the average of each alpha diversity index at even sampling depth of 22,738.

Table S1 Information on DNA extraction and high throughput sequencing libraries of peat soil samples.

No	Sample Name	Concentration of DNA (ng/µl)	Volume (µl)	Number of high quality reads produced
1	MPS (T) Apr-17	63.9	21	29,474
2	MPS (T) Oct-17	23	17	33,315
3	MPS (T) Dec-17	18.5	27	30,510
4	MPS (T) Feb-18	31.4	27	28,618
5	MPS (B) Apr-17	21.5	21	28,016
6	MPS (B) Oct-17	12	18	35,207
7	MPS (B) Dec-17	16.5	29	31,209
8	MPS (B) Feb-18	16.5	27	37,896
9	ABt (T) Apr-17	24.5	21	28,148
10	ABt (T) Oct-17	32	19	31,620
11	ABt (T) Dec-17	13	29	27,340
12	ABt (T) Feb-18	31.4	25	30,598
13	ABt (B) Apr-17	14.5	21	29,620
14	ABt (B) Oct-17	20.5	17	26,067
15	ABt (B) Dec-17	21	28	32,264
16	ABt (B) Feb-18	21.5	25	29,781
17	ABg (T) Apr-17	27.4	21	31,797
18	ABg (T) Oct-17	23	17	29,098
19	ABg (T) Dec-17	16.5	29	33,212
20	ABg (T) Feb-18	33.4	32	37,279
21	ABg (B) Apr-17	19	21	30,786
22	ABg (B) Oct-17	11.5	20	27,929
23	ABg (B) Dec-17	11.5	29	32,742
24	ABg (B) Feb-18	18.5	27	34,786

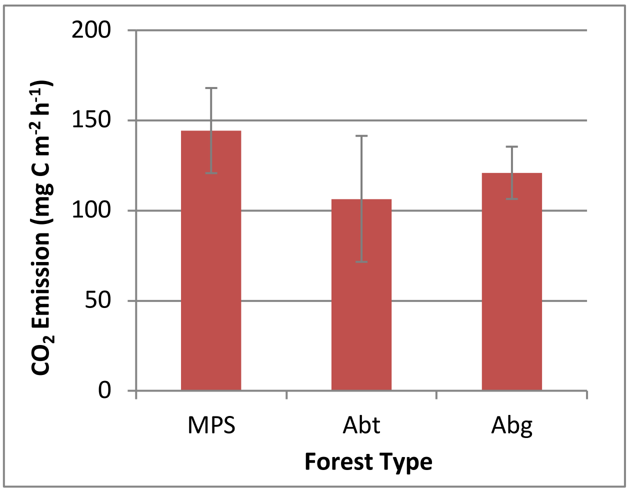

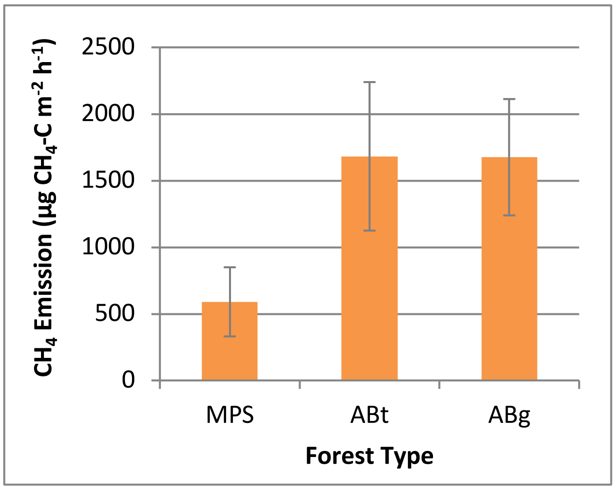

Figure S3 Carbon dioxide and methane emission from soil at MPS, ABt and ABg forests in Maludam National Park, Sarawak. Measurements are taken during the sampling months of April, October, December 2017 and February 2018. Values shown are the mean reading ± SE.

Table S2 Water table measurements at Mixed Peat Swamp, Alan Batu and Alan Bunga forests in Maludam National Park, Sarawak. Measurements are taken during the sampling months of April, October, December 2017 and February 2018. Values shown are the mean reading.

**Month**	**Water Table (cm)**		
	**MPS**	**ABt**	**ABg**
Apr-17	-13.8	-20.1	9.8
Oct-17	-2.2	-10.1	13.9
Dec-17	3.1	1.9	23.3
Feb-18	-2.3	-4.1	26.1
**Average**	**-3.8**	**-8.1**	**18.3**

Table S3. The relative abundance dominant taxa among forest types in different depths. Values of mean ± SE.

Major Taxa	Top (0-20 cm)			Bottom (30-50 cm)		
	MPS	ABt	ABg	MPS	ABt	ABg
Acidobacteria	$29.0±3.3^{a}$	$25.2±2.6^{ab}$	$27.4±2.1^{ab}$	$20.2±2.0^{ab}$	$17.3±2.4^{b}$	$23.6±1.9^{ab}$
Alphaproteobacteria	$21.7±2.0^{ab}$	$18.9±1.8^{b}$	$22.6±1.3^{ab}$	$27.1±1.6^{a}$	$16.3±2.0^{b}$	$17.0±0.7^{b}$
Betaproteobacteria	$3.2±0.7^{a}$	$10.6±2.9^{abc}$	$7.8±2.0^{a}$	$7.0±2.2^{ab}$	$19.7±3.8^{c}$	$15.4±2.0^{b}$
Gammaproteobacteria	$9.6±3.3^{a}$	$5.7±2.3^{a}$	$5.5±1.5^{a}$	$7.2±2.2^{a}$	$6.2±2.6^{a}$	$5.4±2.1^{a}$
Deltaproteobacteria	$5.0±0.7^{ab}$	$7.2±1.4^{ab}$	$3.5±0.3^{b}$	$8.9±1.6^{a}$	$6.9±1.3^{ab}$	$6.6±0.3^{ab}$
Actinobacteria	$4.3±0.5^{a}$	$6.7±1.2^{ab}$	$9.4±1.2^{b}$	$3.8±0.5^{a}$	$3.8±1.1^{a}$	$5.3±0.6^{a}$
Firmicutes	$2.2±1.5^{a}$	$3.9±0.7^{a}$	$3.7±0.9^{a}$	$4.0±1.5^{a}$	$9.8±3.3^{a}$	$6.5±1.3^{a}$
Planctomycetes	$2.2±0.2^{a}$	$3.5±0.5^{a}$	$7.7±1.2^{b}$	$2.7±0.6^{a}$	$2.4±0.7^{a}$	$3.1±0.5^{a}$
Verrucomicrobia	$2.0±0.2^{ab}$	$2.5±0.1^{ab}$	$3.0±0.3^{b}$	$1.6±0.3^{a}$	$1.8±0.2^{a}$	$2.1±0.2^{ab}$
Nitrospirae	$1.7±0.6^{a}$	$0.8±0.2^{a}$	$0.1±0.0^{a}$	$3.9±0.7^{b}$	$1.6±0.5^{a}$	$0.7±0.1^{a}$
Cyanobacteria	$0.7±0.1^{a}$	$0.9±0.1^{a}$	$1.8±0.3^{b}$	$0.60±0.0^{a}$	$0.7±0.1^{a}$	$0.6±0.04^{a}$
Spirochaetes	$0.2±0.02^{a}$	$0.9±0.26^{b}$	$0.2±0.1^{c}$	$0.4±0.05^{a}$	$0.9±0.13^{b}$	$1.2±0.1^{b}$
WPS-2	$1.2±0.3^{ab}$	$0.8±0.2^{ab}$	$1.6±0.1^{b}$	$1.0±0.5^{ab}$	$0.3±0.1^{a}$	$0.4±0.1^{a}$
GAL15	$1.4±0.3^{a}$	$0.1±0.0^{b}$	$0.0±0.0^{b}$	$0.5±0.1^{b}$	$0.2±0.6^{b}$	$0.1±0.0^{b}$
Crenarchaeota	$4.1±0.6^{b}$	$5.5±2.1^{b}$	$1.7±0.2^{a}$	$6.1±0.9^{b}$	$7.5±2.7^{b}$	$5.0±1.2^{b}$
Euryarchaeota	$8.8±1.9^{d}$	$4.0±0.5^{bc}$	$1.4±0.3^{a}$	$2.7±0.2^{b}$	$2.5±0.3^{b}$	$5.1±0.9^{cd}$

Different letters in the same row indicate significant difference at the level of $P<0.05$ based on Tukey's HSD test or pairwise Wilcox test.
